# A Comparison of Health-Related Quality of Life among Normal-Weight, Overweight and Obese Adults in Qazvin Metabolic Diseases Study (QMDS), Iran: Health-Related Quality of Life among Obese Adults

**DOI:** 10.5539/gjhs.v5n3p156

**Published:** 2013-02-26

**Authors:** Azam Ghorbani, Amir Ziaee, Sonia Oveisi, Ahmad Afaghi

**Affiliations:** 1 Instructor of Nursing, Faculty of Nursing, Metabolic Diseases Research Center, Qazvin University of Medical Sciences, Qazvin, Iran; 2 Metabolic Diseases Research Center, Qazvin University of Medical Sciences, Qazvin, Iran

**Keywords:** health-related quality of life, obesity, overweight, BODY MASS INDEX, physical functioning

## Abstract

**Background:**

Obesity is a public health problem that has raised concern worldwide. Numerous epidemiological studies have been showed the relationship between obesity, abdominal fatness and risk of a wide range of illnesses (i.e. diabetes). Obese people experience health-related quality-of-life (HRQL) impairments. The purpose of this study was to evaluate the effect of BMI on Quality of Life, among Normal-Weight, Overweight and Obese adults in Qazvin, Iran.

**Methods:**

This Cross-Sectional study was conducted on 1103 subjects (aged 20-78 years old) from September 2010 to April 2011 in Qazvin, Iran. The study subjects were selected by multistage cluster random sampling method from residents of mindoodar district of Qazvin. Obesity was defined based on Body Mass Index and SF-36 questionnaire was used as measurement instrument for quality of life. Data were analyzed by Chi-square test, ANOVA and MANOVA.

**Results:**

A total of 527 men and 576 women were entered the study. Mean BMI was 25.97 ±4.5 Kg/m^2^. The scores of 6 domains were significantly different between 3 groups of BMI. The differences of physical component summary (PCS) and mental component summary (MCS) scores were also significant between normal weight, overweight and obese subjects (p<0.001 and p<0.025, respectively).

**Conclusion:**

This study underlines the importance of HRQL in overweight and obese individuals. These results suggest that more attention to the obesity and overweight is needed in Iranian population.

## 1. Introduction

With increasing obesity as major health related problem around the world, variety of chronic diseases such as coronary artery disease, hypertension, type II diabetes, and several cancers have become the second leading avoidable cause of mortality in Western countries (Chan & Woo, 2010; Guh et al., 2009; Pi-Sunyer, 2009). Apart from that emerging issue of association between obesity and Health-Related quality of life (HRQL) has gained increasing interest as an outcome measure in clinical setting, public health and among researchers. The phenomenon of quality of life (QOL) is subjective and is reported according to a person’s own experiences and is a combination of physical, psychological, social, emotional, public stress, self-esteem, sexual and work functional domains (Kolotkin & Crosby, 2002a; de Zwaan et al., 2009; Tsai, Yang, Lin, & Fang, 2004). Even, some psychosomatic researchers have focused on pain and mental health issues in HRQL area (Fontaine & Barofsky, 2001; Kolotkin, Crosby, & Williams, 2002b; Saraç et al., 2007). Investigations on the relationship between HRQL and body mass index (BMI) demonstrated that HRQL impairment worsens with increasing obesity (Kolotkin & Crosby, 2002a; Kolotkin et al., 2002b; Fontaine & Bartlett, 1998). The negative effects of obesity on the dimensions of HRQL have been discussed in some reports (Kolotkin & Crosby, 2002a; Kolotkin et al., 2002b; Imai et al., 2008). More over several studies have demonstrated the negative relationship between severity of obesity and well-being and also among obese subjects without chronic disease the physical well-being suppress (Doll, Petersen, & Stewart-Brown, 2000). However in some studies the stronger correlation between obesity and HRQL has been observed among women than men (AHRQ Research Activities, 2007), in others, the domains of HRQL have been influenced by education level (García-Mendizábal et al., 2009).

The HRQL among normal-weight, overweight and obese adults has been investigated in some studies. The results showed that the normal weight individuals had better performance in the physical functioning domain compared to other two groups. Overweight individuals had also better performance than the obese group in this domain (de Zwaan et al., 2009; Tsai et al., 2004; Sirtori et al., 2011; Llaneza et al., 2007; Larsson, Karlsson, & Sullivan, 2002). As regards obesity has become a nationwide epidemic, and this condition is a risk factor for many chronic disease and although several studies have shown the correlation between obesity and poor level of health-related quality of life (HRQL), this is the first study to correlate BMI on quality of life the adult Iranian population; therefore we aimed to evaluate the effect of BMI on quality of life, among normal-weight, overweight and obese adults in Qazvin, Iran.

## 2. Methods

### 2.1 Subjects

The Cross-Sectional study was conducted on 1103 subjects (aged 20-78 years old) from September 2010 to April 2011 in Qazvin (this city is located 150 km northwest of Tehran), Iran. The research project was approved by medical research ethics committee of Qazvin University of Medical Sciences.

The study subjects were selected by multistage cluster random sampling methods from residents of mindoodar district of Qazvin, All households of the district had health profiles at minoodar health center and the sampling unit was the household. The district was divided in to four main cluster according to the population size. Subjects were invited by phone call to attend the study at the health center and after face to face explanation of the study details, they were free to participate. Details of sampling have been reported elsewhere (Ziaee, Esmailzadehha, Ghorbani, & Asefzadeh, 2013). All subjects gave their written informed consent. Inclusion criteria were age ≥ 20 years and no underlying disease. Exclusion criteria included pregnancy, dealing with a mental crisis (such as a family member’s death, mental and physical illness, etc) according to the subjects’ report at the time of study.

### 2.2 Data Collection

Demographic and social data were self-reported in the questionnaire given to the subjects. Two general practitioners filled out the organized questionnaire were prepared from a literature review and face and contact validity of this tool were approved by five experts. Height and weight were measured. Body mass index (BMI) was calculated as weight (kg) divided by the height (m) squared. Normal weight was defined as BMI <25, overweight was defined as 25≤ BMI <30 and obesity was defined as BMI ≥30. To assess subjects’ HRQL, the authors used the short Form of Health Survey (SF-36) questionnaire which has been translated and adapted for Iranian population with the Cranach’s alpha coefficients ranging from 0.77 to 0.90 (alpha = 0.65) and Convergent validity ranging from 0.58 to 0.95 (Montazeri, Goshtasebi, Vahdaninia, & Gandek, 2005). Data were self-reported in the SF-36 questionnaire given to the subjects. The SF-36 questionnaire (Iranian version) which adapted for Iranian population using a well-known tool for assess health-related quality of life. This questionnaire contains 36 questions which measure eight separate dimensions including physical functioning, role-physical, bodily pain, general health, vitality, social functioning, role emotional and mental health. Two aggregate scores reflect physical component summary (PCS) and mental component summary (MCS). Each domain is scored from 0 to 100 where higher scores correspond to a better health-related QOL.

### 2.3 Data Analysis

Statistical analysis: For description of subjects, all variables were presented as frequency, percentage or mean plus or minus standard deviation (SD). Data were analyzed by Chi-square test and analysis of variance (ANOVA). A general lineal model analysis (MANOVA) was also used to compare the scores of BMI groups on the basis of gender. Multivariate logistic regression analyses were run using dichotomized Z-scores on individual domains of SF-36 as dependent variable. This model was adjusted for BMI. Significance level was set as P< 0.05.

## 3. Results

A total of 527 men and 576 women aged 20-78 years were entered the study. Mean BMI was 25.97±4.5 Kg/m^2^. The mean age among normal weight, overweight, and obese subjects were 36.55±12.39, 42.63±8.39 and 42.9±9.06 years, respectively. 464 subjects (42.06%) had normal weight, 451 subjects (40.9%) were overweight and 188 subjects (17.04%) were obese. 39.7% of females were overweight and 24.1% of them were obese. In the male population, 42.1% were overweight and 9.29%were obese.

Socio-demographic characteristics of study subjects are shown in Table 1.in relation to the work status, obese men were more likely to have part (in comparison with normal weight men who had full time job), and the majority of obese women were housewife. The academic education level was higher in normal weight group when compared to overweight and obese groups.

**Table 1:** Socio-demographic characteristics of study subjects

	BMI			Total	P-Value

	<25	25-30	>30		
**Sex**					
Male	256(55.2)	222(49.2)	49(26.1)	527(47.8)	<0.001
Female	208(44.8)	229(50.8)	139(73.9)	576(52.2)	

**Age**					
20-29	155(33.5)	27(6)	12(6.6)	194(17.7)	
30-39	106(22.9)	114(25.3)	47(25.7)	267(24.4)	<0.001
40-49	154(33.3)	232(51.6)	91(49.7)	477(43.6)	
50-59	35(7.6)	62(13.8)	23(12.6)	120(11)	
60>	12(2.6)	15(3.3)	10(5.5)	37(3.4)	

**Education**					
No Formal Education	5(1.1)	11(2.4)	17(9.1)	33(3)	<0.001
<12 years	322(69.5)	377(84)	156(83.9)	855(77.9)	
>12 years	136(29.4)	61(13.6)	13(7)	210(19.1)	

**Work Status**					
Employed	213(46.2)	157(34.9)	32(17.3)	402(36.7)	
Unemployed	46(10)	7(1.6)	1(0.5)	54(4.9)	<0.001
Housewife	132(28.6)	202(44.9)	130(70.3)	464(42.3)	
Pensioner(Retired)	70(15.2)	84(18.7)	22(11.9)	176(16.1)	

Data are shown as Number (percent).

**Figure 1:**
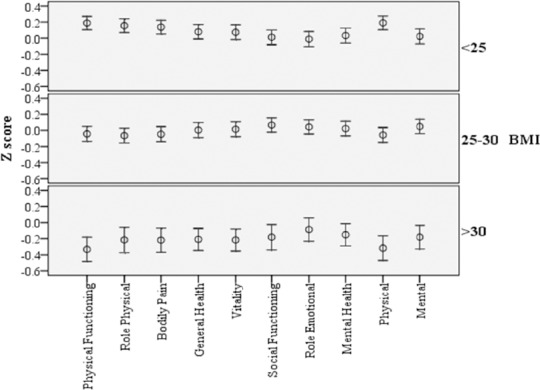
Z-score on short Form-36 in relation to normal weight, overweight and obesity. Data are presented as means and 95% confidence intervals

The scores of 6 domains were significantly different between 3 groups of BMI (Table 2). From a total of eight domain studied, six of them were different among the groups based on BMI (physical functioning, role physical, bodily pain, general health, vitality and social functioning). The differences of PCS and MCS scores were also significant between normal weight, overweight and obese subjects (p<0.001 and p<0.025, respectively). Comparing the scores of 8 domain of HRQL of normal weight, overweight, and obese groups showed that among all domains, only the role physical domain showed significant difference between two sexes (p<0.001). Among females the PCS score was significantly different between normal weight, overweight and obese subjects (p<0.001). General linear model analyses revealed that the BMI and sex had significant effect in role physical domain (P<0.001). The Z-scores on SF-36 domains, showed the impairment of HRQL in comparison with BMI and reflecting that HRQL was particularly poor in physical component summary (PCS) in obese subjects. SF-36 Z-scores in three groups are summarized in Figure 1.

**Table 2:** SF-36 scores of health-related quality of life in 3 groups of study population

Domains	Total	BMI			F	P-value

		<25	25-30	>30		
**Physical Functioning**	79.4±21.7	83.51±19.75	74.48±22.05	72.23±22.59	19.84	<0.001
**Role Physical**	69.5±34.6	74.94±32.59	67.29±34.44	62.10±38.03	11.128	<0.001
**Bodily Pain**	63.8±21.4	66.76±20.09	62.79±21.88	59.14±22.40	9.53	<0.001
**General Health**	62.2±17.7	63.65±17.56	62.31±18.09	58.54±16.72	5.61	0.004
**Vitality**	61.4±18.2	62.78±18.22	61.70±18.43	57.5±17.27	5.73	0.003
**Social Functioning**	74.9±21.4	75.21±21.35	76.38±20.53	71.07±23.31	4.13	0.016
**Role Emotional**	66.7±37.6	66.30±38.78	68.36±36.023	63.47±38.09	1.15	0.315
**Mental Health**	66.5±17.9	67.12±18.34	66.92±17.81	63.80±17.34	2.51	0.081
**Physical (PCS)**	68.77±18.18	72.22±16.90	67.72±18.20	63.0±19.36	19.17	<0.001
**Mental(MCS)**	67.42±19.16	67.85±19.53	68.34±18.55	63.96±19.44	3.71	0.025

## 4. Discussion

Our findings showed that overweight and obese subjects suffered from poor HRQL and had lower scores than normal weight subjects. This study revealed that HRQL of overweight and obese subjects in 6 domains were significantly different from normal weight subjects. These differences were in physical functioning, role physical, bodily pain, general health, vitality and social functioning.

The current findings were similar to previous reports (Tsai et al., 2004; Mannucci et al., 2010; Williams, Wake, Hesketh, Maher, & Waters, 2005; Vasiljevic et al., 2008; Saraç et al., 2007). Vasilgeic et al. (2008) reported that in Belgrad population the normal weight subjects compared to overweight and obese subjects had significant differences in 7 domains’ scores, while in a study conducted in Turkey by Sarac et al. (2007), these differences were reported in 6 domains.

Considering that overweight and obesity affect physical health and limit the daily activity of subjects (Alley & Chang, 2007), increasing BMI causes HRQL decrease in physical domains (Larsson et al., 2002; Vasiljevic et al., 2008; Saraç et al., 2007; Alley & Chang, 2007; Wiczinski, Döring, John, von Lengerke & KORA Study Group, 2009). Although in our study increasing BMI resulted in suppression of all domains of HRQL scores, this negative effect was more severe in physical domains. Several reports have discussed the relationship between increasing BMI and complaining from physical disorders and incompetency (Ucan & Ovayolu, 2010; Dinç et al., 2006; Kolotkin, Meter, & Williams, 2001). Other studies found that, increasing severity of obesity resulted in reducing general health (Richards, Adams, & Hunt, 2000) and increasing number of physical disorders (de Zwaan et al., 2009; Sirtori et al., 2011). Our findings is similar to previous mentioned studies and the significant differences in scores of bodily pain, role physical, and general health domains between normal weight and obese groups confirm these findings.

Wen-Ling Tsai also confirmed the relationship between increase in BMI and physical functioning, bodily pain, physical role limitations in women (Tsai, 2004). Moreover, in a study conducted in Korean adults by Song, obese women had lower QOL than men (Song et al., 2010). Our results showed close relationship between QOL and physical domains that corresponds with previous reports (de Zwaan et al., 2009; Doll et al., 2000; Fontaine et al., 1998). It seems that the physical QOL domains will progressively impair with increasing BMI and age. We can conclude that overweight and obesity have a major role in the poor HRQL (de Zwaan et al., 2009).

Our results showed significant differences between physical domains and education in women. Obese women with lower education had lower physical QOL, which was similar with the results in Spanish women (García-Mendizábal et al., 2009). On the other hand, researchers emphasized that these differences in women with higher level of education were not significant. We can conclude that obesity and low education could be one of the most important predictor of QOL in both physical and psychosocial domains (de Zwaan et al., 2009).

The present study demonstrated significant relationship between BMI and some mental domains of QOL. Weak relationship between mental domains’ scores and BMI in obese and non-obese subjects has been reported in many studies (de Zwaan et al., 2009; Doll et al., 2000; Saraç et al., 2007). Although the impact of overweight and obesity on mental health is controversial (García-Mendizábal et al., 2009); some reports state that obesity cannot be associated with an increase in mental disorders (Friedman & Brownell, 1995). According to available documents and the importance of women’s role in family, special consideration should be placed on future health plans; because the risk of mental health problems are higher in obese subjects than in normal-weight and overweight population. In addition the risk of depression and suicidal thoughts are higher in women (de Zwaan et al., 2009; Saraç et al., 2007; Roberts, Kaplan, Shema, & Strawbridge, 2000).

In 2010, the World Health Organization reported about the Changing pattern of diseases towards chronic and non-communicable diseases and prevalence of obesity in the world (World Health Organization Global database on body mass index, 2010). In addition, obesity has a negative impact on health issue of population (especially women of reproductive age) and physical problems such as infertility, a variety of non-communicable diseases, cancer and other conditions (World Health Organization Global database on body mass index, 2010; Chescheir, 2011) and reduces physical and mental QOL. Preparation of health goals, action plans and multi disciplinary approaches (Montazeri et al., 2005) with regard to the social and demographic characteristics of each region are required (Musaiger, 2011).

The strengths of this study are the inclusion of a large sample, the direct measurement of anthropometric indices, this is the first study correlating body mass index (BMI) and quality of life of the adult Iranian population, using a well-known questionary (SF-36), which has translated and adapted for Iranian population. Lack of detailed information about undiagnosed physical problems and social and economic issues of the subjects’ life are limitations of the current study.

In summary, assessment of quality of life had been considered in different communities. The study showed that the overweight and obese subjects suffer from poor HRQL, as the increase in BMI had lowered the domains of HRQL. The physical domain was more severe affected by obesity. The authors suggest the use of HRQL as outcome measurement in treatment programs. Based on results, more attention to quality of life in health promotion programs in obese and overweight groups in necessary for physical and social well-being Iranian population.
